# Active site motifs of the Nem1 protein phosphatase confer its function in yeast lipid synthesis

**DOI:** 10.1016/j.jbc.2026.113385

**Published:** 2026-08-04

**Authors:** Emily R. Bostrom, Ruta Jog, Geordan J. Stukey, Gil-Soo Han, George M. Carman

**Affiliations:** Department of Food Science and the Rutgers Center for Lipid Research, Rutgers University, New Brunswick, New Jersey, USA

**Keywords:** phosphatidate, diacylglycerol, triacylglycerol, Nem1-Spo7 protein phosphatase, Pah1 phosphatidate phosphatase, haloacid dehalogenase-like domain

## Abstract

The Nem1-Spo7 protein phosphatase complex plays a pivotal role in the lipid metabolism of *Saccharomyces cerevisiae* by activating Pah1, which catalyzes the dephosphorylation of phosphatidate to produce diacylglycerol for the synthesis of triacylglycerol and the phospholipids phosphatidylcholine and phosphatidylethanolamine. As the catalytic subunit, Nem1 dephosphorylates Pah1 to facilitate its membrane localization for phosphatidate phosphatase activity. In this study, we investigated the functional importance of the Nem1 residues (Asp-257, Asp-259, Thr-261, Thr-319, Lys-360, Asp-386, and Asn-387) conserved in the four active site motifs of the haloacid dehalogenase (HAD)-like domain. The effects of alanine mutations of the active site residues on Pah1 dephosphorylation and associated functions in lipid synthesis were examined. Our results showed that the Nem1 active site mutants are catalytically inactive without a defect in protein stability, complex formation with Spo7, and recruitment of Pah1 to the membrane. Cells expressing the Nem1 active site mutants are defective in the activation of Pah1 for triacylglycerol synthesis and the regulation of phosphatidylserine synthase Cho1 expression. These findings demonstrate that the active site motifs of Nem1 are important for catalytic activity, which is required for the full activation of Pah1 and its role in cellular lipid homeostasis.

In *Saccharomyces cerevisiae*, the *PAH1*-encoded phosphatidic acid (PA) phosphatase is a key regulator in lipid synthesis (1, 2, 3). By catalyzing the dephosphorylation of PA to generate diacylglycerol (DAG) (4, 5), Pah1 reciprocally controls the levels of the key intermediates in the biosynthetic pathways to synthesize membrane phospholipids and the storage lipid triacylglycerol (TAG) (6, 7). PA is positioned at a metabolic branch point: it is dephosphorylated to DAG for the production of TAG, or converted to CDP-DAG for the *de novo* synthesis of phospholipids in subsequent biosynthetic reactions (CDP-DAG → phosphatidylserine → phosphatidylethanolamine → phosphatidylcholine) (6, 7). When the CDP-DAG pathway is defective (*e.g.*, loss of phosphatidylserine synthase (8, 9)), phosphatidylcholine and phosphatidylethanolamine can be synthesized from the DAG by the CDP-choline and CDP-ethanolamine branches, respectively, of the Kennedy pathway (6, 7).

In addition to being a metabolic intermediate, PA is a key component in the Henry (Opi1/Ino2-Ino4) regulatory circuit for the sequestration of the Opi1 transcriptional repressor at the nuclear/ER membrane (10, 11, 12). The PA-associated Opi1, which is prevented from entering the nucleus, cannot inhibit the function of Ino2-Ino4 activator complex that interacts with the UAS_INO_ element in the promoters of phospholipid synthesis genes (6, 7, 10, 12). When PA levels drop, Opi1 is free to enter the nucleus and bind to Ino2, effectively repressing the expression of the phospholipid synthesis genes (6, 7, 10, 12). Consequently, when Pah1 activity is high (*e.g.*, in stationary phase), the metabolic flux is directed away from phospholipid synthesis and toward the synthesis of TAG. Conversely, when Pah1 activity is low (*e.g.*, in exponential phase or *pah1*Δ mutant), phospholipid synthesis is favored at the expense of TAG (1, 13, 14, 15).

The Nem1 (catalytic subunit)-Spo7 (regulatory subunit) phosphatase complex is the vital activator of Pah1, whose cellular function is strictly controlled by its membrane localization (3, 16, 17, 18). Pah1 is phosphorylated by an assortment of protein kinases that include the cyclin-dependent protein kinases Pho85-Pho80 (19) and Cdc28 (20), protein kinases A (21) and C (22), casein kinases I (23) and II (24), glycogen synthase kinase Rim11 (25), and septin-associated protein kinase Hsl1 (26). While Pah1 phosphorylation, particularly by Pho85-Pho80, sequesters the enzyme in the cytosol (19), it also protects the enzyme from degradation by the 20S proteasome (27, 28). The phosphorylation also attenuates its PA phosphatase activity (19). For its catalytic function, Pah1 as a phosphorylated form in the cytosol is recruited and dephosphorylated by Nem1-Spo7 at the nuclear/ER membrane, and the dephosphorylated form associates with the membrane using the N-terminal amphipathic helix (17, 18, 29, 30). While multiple protein kinases are involved in the phosphorylation of Pah1, Nem1-Spo7 is the only protein phosphatase responsible for the dephosphorylation of the enzyme (31) (Fig. 1).

**Figure 1:**
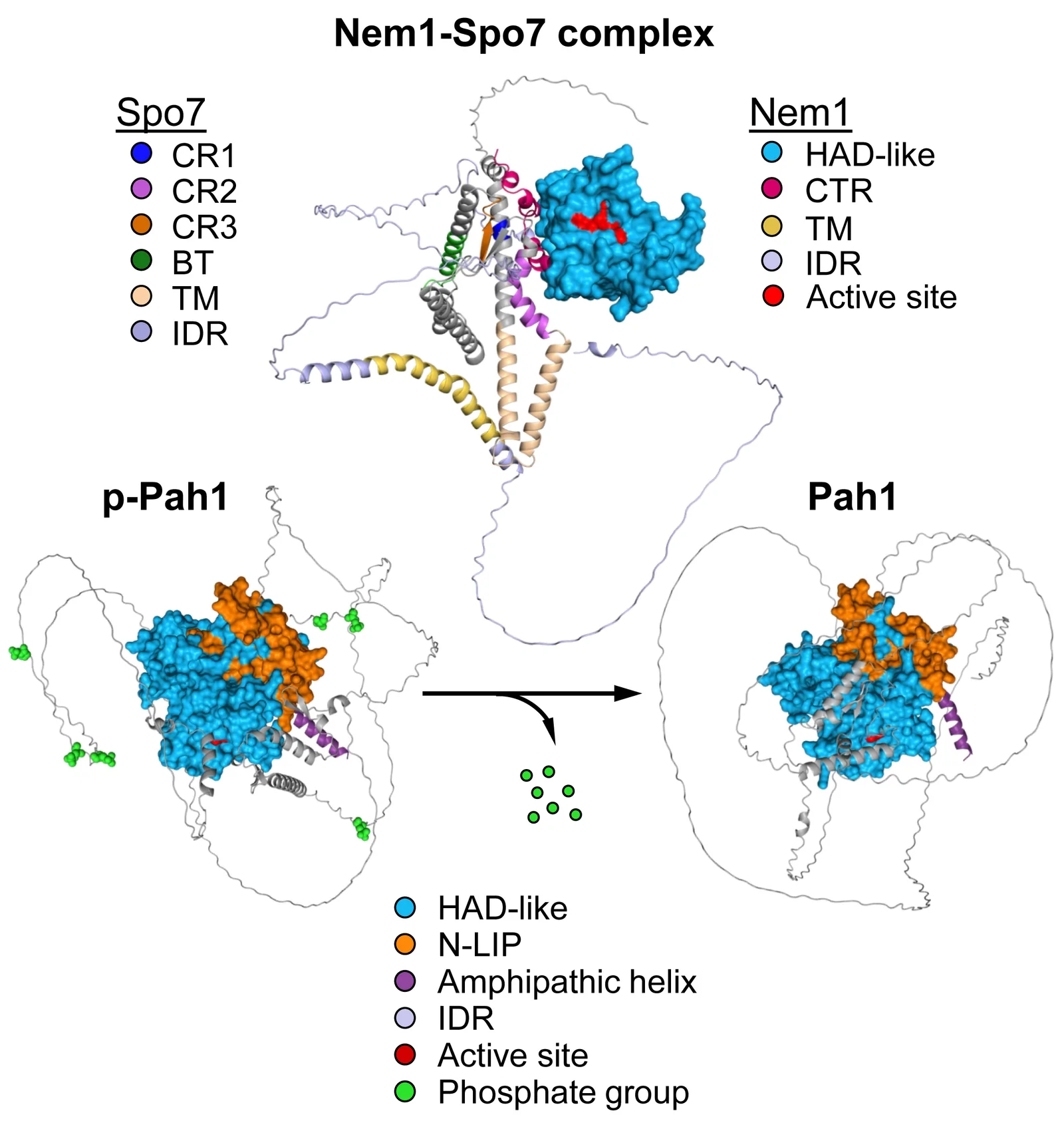
**Reaction catalyzed by the Nem1-Spo7 complex.** The Nem1-Spo7 complex catalyzes the dephosphorylation of Pah1. The predicted AlphaFold (56) structures of the Nem1-Spo7 complex and the phosphorylated and dephosphorylated forms of Pah1 are visualized by the PyMol program. The phosphorylations mediated by Pho85-Pho80 (19) are denoted by *green* circles. The domains/regions of Nem1, Spo7, and Pah1 are indicated in the legend. The Pah1 acidic tail is not shown in the model as it is positioned behind the catalytic core. BT, basic tail; CR, conserved region; CTR, C-terminal region; IDR, intrinsically disordered region; TM, transmembrane.

The haloacid dehalogenase (HAD)-like domain of Nem1 is responsible for its catalytic function (16), whereas its C-terminal region (32) interacts with the conserved regions CR1-CR3 of Spo7 (33, 34) to form a complex. The basic tail of Spo7 (35) is responsible for the priming interaction with the acidic tail of Pah1 (36). The transmembrane regions of Nem1 and Spo7 anchor the complex to the nuclear/ER membrane (16). Mutations in any of these domains/regions obviate the function of the Nem1-Spo7/Pah1 phosphatase cascade (32, 33, 34, 36). The complex itself is subject to phosphoregulation. For example, phosphorylations of Nem1 and Spo7 by protein kinase C stimulate the phosphatase activity (37), whereas their phosphorylations by protein kinase A are inhibitory to the activity (38).

Like Pah1 (39, 40), Nem1 (16) is a Mg^2+^-dependent phosphatase (30) that belongs to the HAD-like superfamily of hydrolases characterized by the D*X*D*X*(T/V) motif that catalyze a phosphoryl transfer reaction (41, 42, 43, 44, 45, 46). This superfamily encompasses a broad range of phosphatases with diverse substrate specificities (41). HAD-like enzymes contain the Rossmann-like fold that features a central β-sheet composed of 5 to 7 parallel β-strands surrounded by α-helices (43, 47, 48) (Fig. 2). The HAD-like domain contains four active site motifs (41, 43, 44, 45, 46, 47, 49, 50, 51, 52, 53, 54, 55). Motif I (D*X*D*X*(T/V)) is found at the end of β-strand 1. In motif I, the first Asp acts as a nucleophile, forming a phosphoryl-aspartate intermediate; the second Asp deprotonates a water molecule to facilitate hydrolysis of this intermediate; and the Thr/Val provides a backbone carbonyl to coordinate the essential Mg^2+^ (42, 44, 53). Motif II contains a Thr or Ser residue at the end of β-strand 2, and Motif III features a conserved Lys residue within an α-helix surrounding the central β-sheet with its side chain pointing towards the active site (Fig. 2). Motif IV is found at the end of β-strand four and is broadly represented as G(D/N)*XXX*D or D(D/N) (43). Motifs II and III are thought to stabilize the reaction intermediate (43), and motif IV, along with Thr/Val of motif I, coordinates the Mg^2+^ (43, 50).

**Figure 2:**
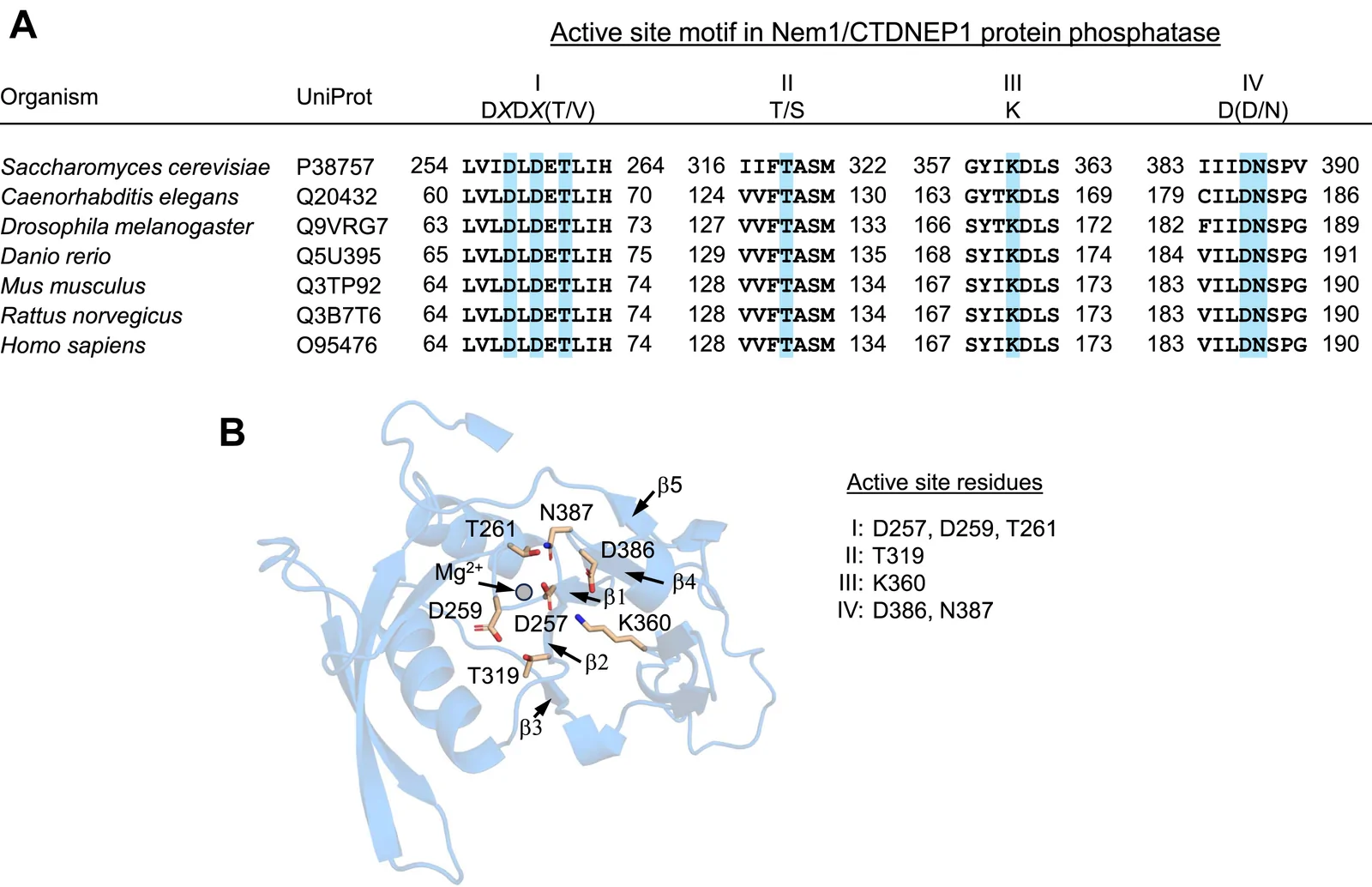
**HAD-like domain active site motifs of Nem1/CTDNEP1 protein phosphatases and predicted Nem1 active site architecture.***A*, clustal Omega alignment of HAD-like domain motifs in Nem1/CTDNEP1 protein phosphatases with their UniProt accession numbers. Residue numbers are indicated at the start and end of the aligned sequences. The conserved residues of each motif are highlighted in *blue color*. *B*, AlphaFold (56) prediction of the Nem1 active site visualized by the PyMol program. The conserved residues of active site motifs I-IV, and β-strands are indicated. Mg^2+^ is shown by the *gray circle*.

The sequence analysis of Nem1 and its structure predicted by AlphaFold (56) indicated that the conserved active site residues are Asp-257, Asp-259, and Thr-261 (motif I), Thr-319 (motif II), Lys-360 (motif III), and Asp-386 and Asn-387 (motif IV) (Fig. 2). While the importance of the D*X*D*X*(T/V) motif to Nem1 phosphatase activity has been examined in a preliminarily manner (16), the importance of motifs II-IV remain uncertain. In this work, we show that all four motifs are required for the dephosphorylation of Pah1, and this advances our understanding of the catalytic mechanism of the Nem1 phosphatase and its role in lipid synthesis and regulation.

## Results

### Conserved residues of Nem1 active site motifs are required for catalytic activity

**Figure 3:**
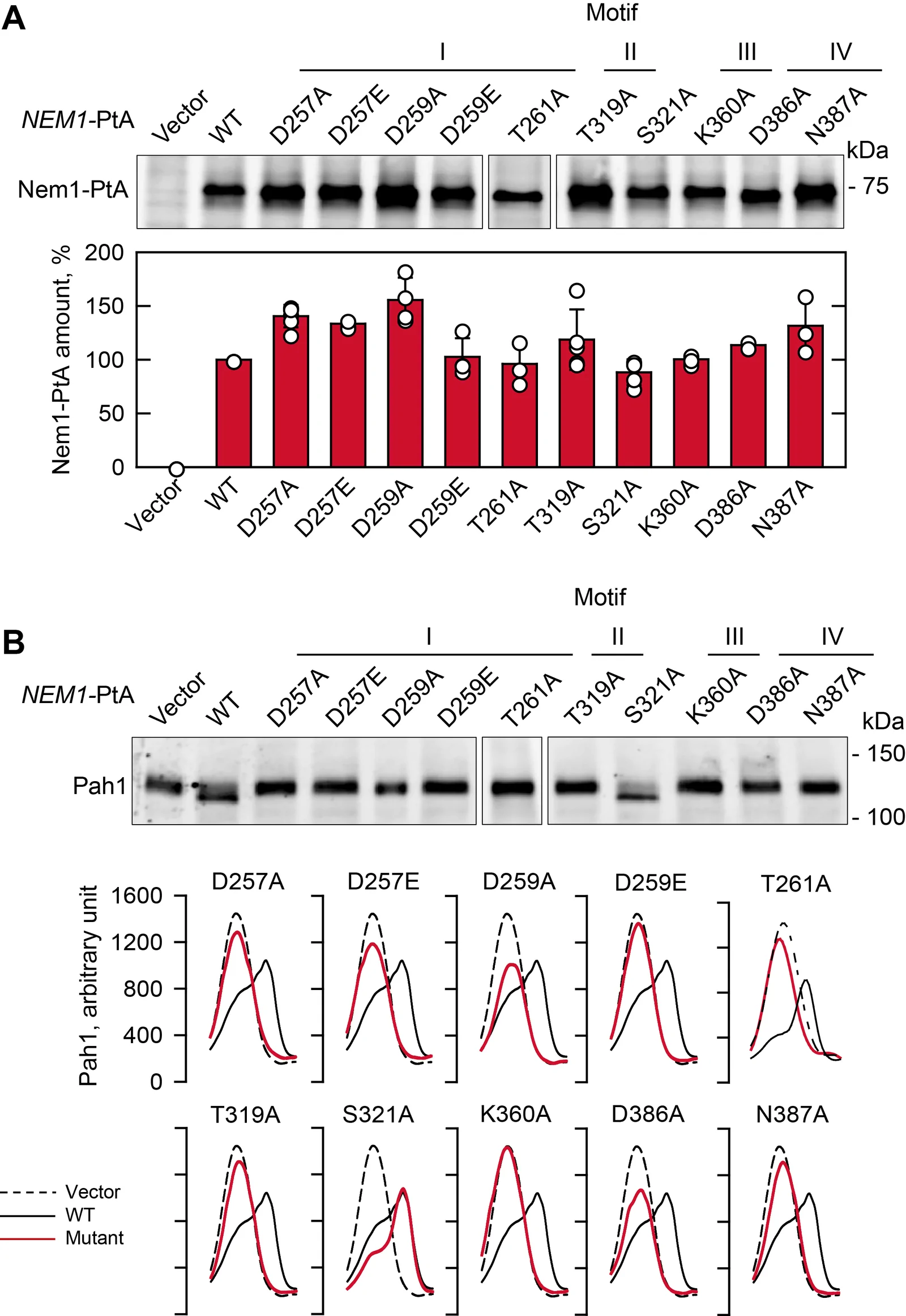
**Nem1 active site mutations abolish the Nem1-Spo7 complex-mediated dephosphorylation of Pah1.***A*, (*upper*), the *nem1*Δ *spo7*Δ *pah1*Δ (GHY85) cells expressing plasmids YCplac111 (vector) or YCplac111-*GAL1/10-NEM1*-PtA (WT or mutant derivatives) and pRS314-*GAL1/10-SPO7* were incubated at 30 °C for 14 h in SC-Leu-Trp medium with 2% galactose and 1% raffinose. Following the induction, membranes (30 μg) isolated from cell extracts were subjected to SDS-PAGE (12.5% polyacrylamide gel), transferred to polyvinylidene difluoride membrane, and probed for Nem1-PtA using rabbit anti-protein A antibody. Sample loading was normalized to total membrane protein. The positions of Nem1-PtA and molecular mass standards are indicated. The data shown in the blot are representative of at least three replicate experiments. *A*, (*lower*), the relative amounts of the WT and mutant forms of Nem1-PtA were determined by ImageQuant analysis of at least three independent experiments ± SD (*error bars*). The individual data points are shown. *B*, (*upper*), phosphorylated Pah1 (5 ng) was incubated for 25 min with membranes (20 μg) prepared from *nem1*Δ *spo7*Δ *pah1*Δ (GHY85) cells expressing plasmids YCplac111 (vector) or YCplac111-*GAL1/10-NEM1*-PtA (WT or mutant derivatives) and pRS314-*GAL1/10-SPO7* under the assay conditions for Nem1-Spo7 phosphatase activity (30). Following the incubation, the reaction mixtures were resolved by SDS-PAGE (6% polyacrylamide gel), transferred to polyvinylidene difluoride membrane, and probed with anti-Pah1 antibody. The positions of Pah1 and molecular mass standards are indicated. T261A samples were electrophoresed on individual gels under identical conditions. The figure incorporates spliced lanes to maintain the positional order of the motif residues. *B*, (*lower*), the signal intensities of Pah1 along its migration in the region between the slower migrating phosphorylated and faster migrating dephosphorylated forms were measured using the line graph function of ImageQuant software. The densitogram of Pah1 mobility after incubation with membranes expressing the vector (*black dashed line*), WT (*black line*) and mutant (*red line*) forms of Nem1 is shown. The data shown is representative of triplicate experiments.

Nem1, in complex with Spo7, serves as the catalytic subunit to dephosphorylate Pah1 (17, 34, 35). We examined the importance of the putative active site residues in the Nem1 HAD-like domain (Fig. 2) by substituting them with the alanine residue (Table 1). Motif I residues Asp-257 and Asp-259 were also mutated to glutamate to assess the importance of the acidic residues. Ser-321, a non-active site residue, was included in the analysis as a control. *NEM1*-PtA and its mutant derivatives and *SPO7* were overexpressed under the galactose-inducible *GAL1/10* promoter (Table 1). Western blot analysis showed that the amounts of the mutant proteins were comparable to, or exceeded, that of the WT (Fig. 3*A*). Pah1, purified from yeast as a phosphorylated form (57), was incubated with the membranes prepared from the *nem1*Δ *spo7*Δ *pah1*Δ cells overexpressing Nem1 (WT or mutant derivatives) and Spo7, and then examined for its electrophoretic mobility in SDS-PAGE (30, 33, 34, 35). As expected (30), Pah1 showed faster migration when it was incubated with the membranes containing WT Nem1 and Spo7 (Fig. 3*B*). The decreased abundance of faster-migrating Pah1 is attributed to its dephosphorylation, which renders the enzyme susceptible to proteolytic degradation (27, 28). Similar changes in Pah1 mobility were shown by the membrane of S321A, a non-active site mutant of Nem1. However, the Nem1 active site mutants did not affect the electrophoretic mobility and abundance of Pah1 (Fig. 3*B*), indicating that they are defective in dephosphorylation of Pah1. The charge-mimicking mutations in catalytic residues (*i.e.*, D257E and D259E) abolished the enzyme activity, suggesting the importance of spatial geometry in the active site residues.

**Table 1:** Strains and plasmids used in this study

Strain or plasmid	Relevant characteristics	Source or Reference
Strain		
*E.coli*		
DH5α	F^-^ ϕ80d*lacZ*ΔΜ15Δ (*lacZYA*-*argF*)U169 *deoR rec*A1 *end*A1 *hsd*R17 (*r*_k_^-^*m*_*k*_^+^) *pho*A *sup*E44 λ^−^*thi-*1 *gyr*A96 *rel*A1	(90)
*S*. *cerevisiae*		
RS453	*MAT*a *ade2-1 his3-11*,*15 leu2-3112 trp1-1 ura3-52*	(104)
Derivatives		
WMY161	*nem1*Δ*::URA3*	(38)
SS1010	*nem1*Δ*::HIS3 spo7*Δ*::HIS3*	(16)
GHY85	*nem1*Δ*::HIS3 spo7*Δ*::HIS3 pah1*Δ*::natMX4*	(34)
SS1132	*nem1*Δ*::HIS3 pah1*Δ*::TRP1*	(20)
Plasmid		
YCplac111	Low-copy number *E. coli*/yeast shuttle vector with *LEU2*	(88)
Derivatives		
YCplac111-*NEM1*	*NEM1* with native promoter in YCplac111	(32)
YCplac111-*NEM1*-D257A	*NEM1* with the D257A mutation	This study
YCplac111-*NEM1*-D257E	*NEM1* with the D257E mutation	This study
YCplac111-*NEM1*-D259A	*NEM1* with the D259A mutation	This study
YCplac111-*NEM1*-D259E	*NEM1* with the D259E mutation	This study
YCplac111-*NEM1*-T261A	*NEM1* with the T261A mutation	This study
YCplac111-*NEM1*-T319A	*NEM1* with the T319A mutation	This study
YCplac111-*NEM1*-S321A	*NEM1* with the S321A mutation	This study
YCplac111-*NEM1*-K360A	*NEM1* with the K360A mutation	This study
YCplac111-*NEM1*-D386A	*NEM1* with the D386A mutation	This study
YCplac111-*NEM1*-N387A	*NEM1* with the N387A mutation	This study
YCplac111-*GAL1/10*-*NEM1*-PtA	*NEM1*-PtA with *GAL1/10* promoter in YCplac111	(16)
YCplac111-*GAL1/10*-*NEM1*-D257A-PtA	*NEM1*-PtA with the D257A mutation	This study
YCplac111-*GAL1/10*-*NEM1*-D257E-PtA	*NEM1*-PtA with the D257E mutation	This study
YCplac111-*GAL1/10*-*NEM1*-D259A-PtA	*NEM1*-PtA with the D259A mutation	This study
YCplac111-*GAL1/10*-*NEM1*-D259E-PtA	*NEM1*-PtA with the D259E mutation	This study
YCplac111-*GAL1/10*-*NEM1*-T261A-PtA	*NEM1*-PtA with the T261A mutation	This study
YCplac111-*GAL1/10*-*NEM1*-T319A-PtA	*NEM1*-PtA with the T319A mutation	This study
YCplac111-*GAL1/10*-*NEM1*-S321A-PtA	*NEM1*-PtA with the S321A mutation	This study
YCplac111-*GAL1/10*-*NEM1*-K360A-PtA	*NEM1*-PtA with the K360A mutation	This study
YCplac111-*GAL1/10*-*NEM1*-D386A-PtA	*NEM1*-PtA with the D386A mutation	This study
YCplac111-*GAL1/10*-*NEM1*-N387A-PtA	*NEM1*-PtA with the N387A mutation	This study
pRS314	Low-copy number *E. coli*/yeast shuttle vector with *TRP1*	(89)
Derivative		
pRS314-*GAL1/10-SPO7*	*SPO7* with *GAL1/10* promoter in pRS314	(38)
GH452	*PAH1-*PtA with *GAL1* promoter in pYES2	(57)

### Nem1 active site mutations do not affect the complex formation with Spo7

**Figure 4:**
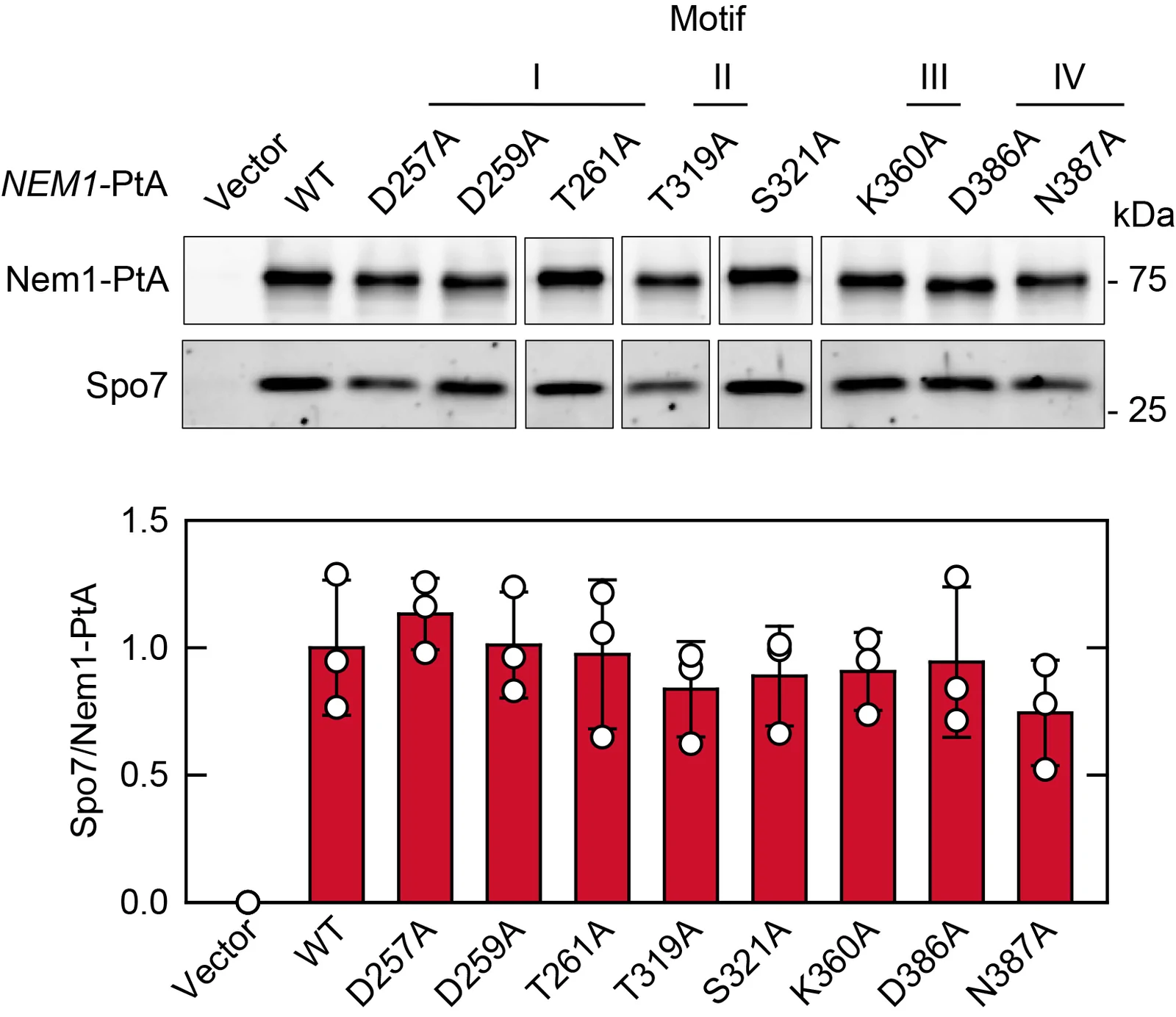
**Nem1 active site mutations do not impair complexation with Spo7.***Upper*, cell extracts were prepared from *nem1*Δ *spo7*Δ *pah1*Δ (GHY85) cells co-expressing plasmids YCplac111, YCplac111-*GAL1/10*-*NEM1*-PtA (WT or mutant derivatives) and pRS314-*GAL1/10-SPO7*. The cell extracts were used for the isolation of protein A-tagged Nem1 proteins by IgG-Sepharose affinity chromatography. The affinity-purified Nem1 preparations were resolved by SDS-PAGE (10% polyacrylamide gel) and transferred to a polyvinylidene difluoride membrane. Sample loading was normalized to the elution volume of purified Nem1-PtA. The polyvinylidene difluoride membrane was split into Nem1-PtA and Spo7 portions, and probed with rabbit anti-protein A and rabbit anti-Spo7 antibodies, respectively. The formation of the Nem1-Spo7 complex was scored by the presence of Spo7 in the affinity-purified Nem1 preparation (30). The positions of Nem1-PtA, Spo7, and molecular mass standards are indicated. T261A, T319A, and S321A samples were electrophoresed on individual gels under identical conditions. The figure incorporates spliced lanes to maintain the positional order of the motif residues. Data shown are representative of triplicate experiments. *Lower*, the relative amounts of Nem1-PtA and Spo7 were determined by ImageQuant analysis and the data for the ratio of Spo7 to Nem1-PtA are means ± SD (*error bars*) from triplicate experiments. The value for the WT complex was set at 1.0. The individual data points are shown.

In the Nem1-Spo7 complex, Spo7 serves as a regulatory subunit required for Nem1 catalytic activity (16, 32, 33, 34). Together, they recruit and dephosphorylate Pah1 at the nuclear/ER membrane, thereby stimulating its function (17, 18, 29, 30, 31, 36). To confirm that the active site mutations did not disrupt complex formation, Nem1-PtA was purified by affinity chromatography with IgG Sepharose from *nem1*Δ *spo7*Δ *pah1*Δ cells expressing the plasmids with *GAL1/10*-*NEM1-*PtA (WT or mutant derivative) and *GAL1/10*-*SPO7*. The Western blot analysis showed that Spo7 co-purified with both WT Nem1-PtA and all tested active site mutants (Fig. 4), demonstrating that the observed loss of Nem1 phosphatase activity on Pah1 resulted from catalytic impairment rather than a failure to assemble the complex.

### Nem1 active site mutations do not affect the association of Pah1 with the membrane

**Figure 5:**
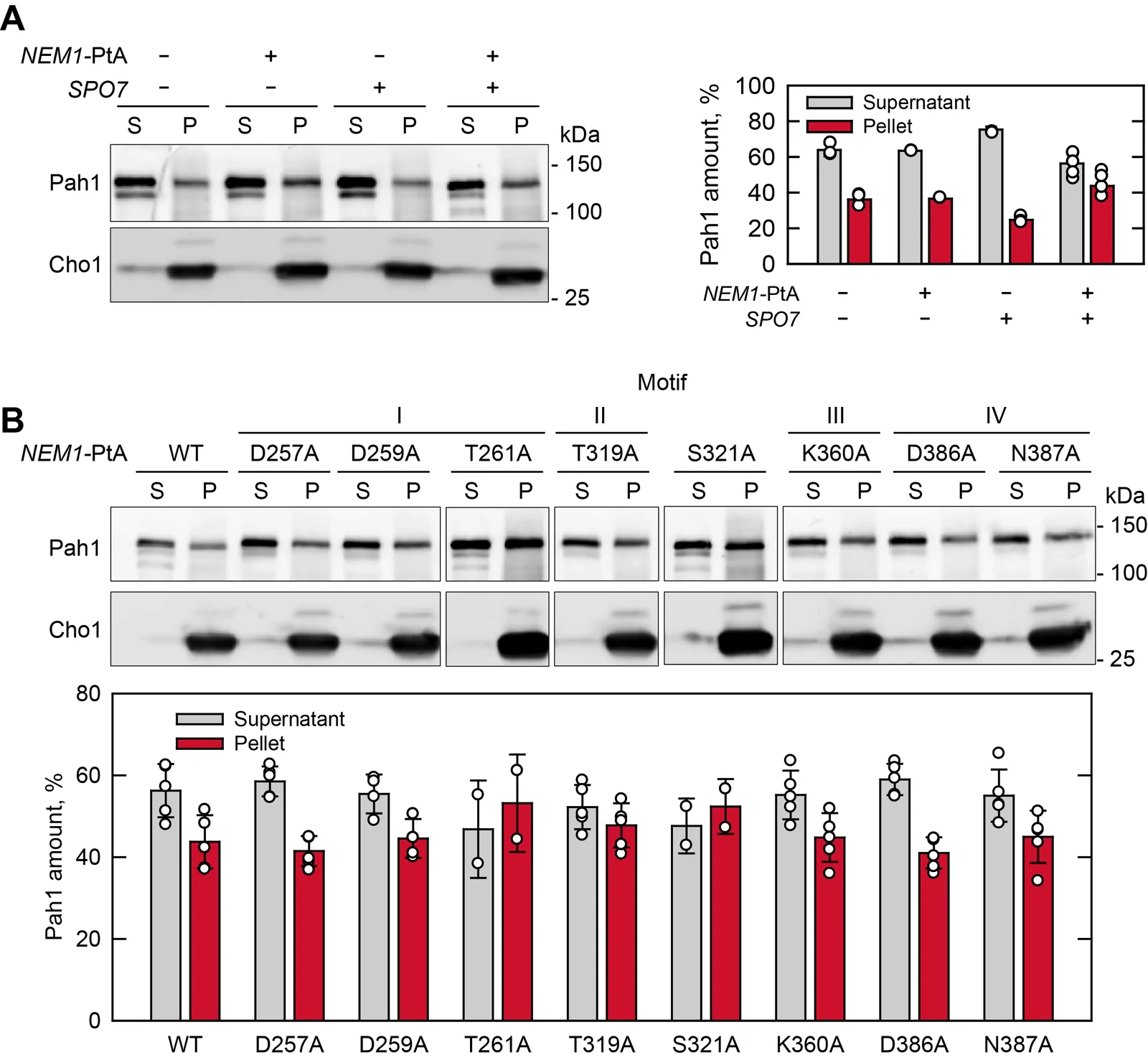
**Nem1 active site mutations do not impair the Nem1-Spo7-mediated translocation of Pah1 to membranes.** Purified phosphorylated Pah1 (20 ng) was incubated for 20 min at 30 °C in a total volume of 20 μl with the membranes (30 μg) prepared from *nem1*Δ *spo7*Δ *pah1*Δ (GHY85) cells expressing the YCplac111-*GAL1/10*-*NEM1*-PtA and/or pRS314-*GAL1/10-SPO7* as denoted by the + and - symbols (*A*). Membranes were also prepared from *nem1*Δ *spo7*Δ *pah1*Δ (GHY85) cells co-expressing YCplac111-*GAL1/10*-*NEM1*-PtA (WT or mutant derivatives) and pRS314-*GAL1/10-SPO7* (*B*). Following the incubation, the mixture was centrifuged at 100,000*g* for 1 h at 4 °C. The membrane pellet (*P*) was resuspended to the volume of the supernatant (*S*), and equal proportions of the pellet and supernatant fractions were resolved by SDS-PAGE (10% polyacrylamide gel) and transferred to a polyvinylidene difluoride membrane, followed by probing with rabbit anti-Pah1 and rabbit anti-Cho1 (ER membrane marker) antibodies. The positions of Pah1, Cho1, and molecular mass standards are indicated. T261A, T319A, and S321A samples were electrophoresed on individual gels under identical conditions. The figure incorporates spliced lanes to maintain the positional order of the motif residues. The data shown in the blots are representative of at least three independent experiments. The relative amounts of Pah1 in the supernatant and pellet fractions were determined by ImageQuant analysis. The data (*A*, *right*; *B*, *lower*) are means ± SD from at least three independent experiments. The individual data points are also shown.

Since Nem1-Spo7 recruits Pah1 to the nuclear/ER membrane (17, 19, 20, 30, 34), we investigated whether the Nem1 active site mutations affect this function of the complex. For this experiment, purified phosphorylated Pah1 was incubated with Pah1-free membranes with overexpressed levels of Nem1-PtA and Spo7, and then fractionated into supernatant and pellet fractions for detection of its membrane association (35, 57). Enrichment of the membrane fraction was confirmed by Western blot analysis of the ER marker Cho1 (Fig. 5, *A* and *B*). In control experiments, most Pah1 remained in the soluble fraction when incubated with membranes lacking the complex or either one of its components (Fig. 5*A*). When the complex was present in the membranes, the amount of Pah1 was reduced in the soluble fraction with a concomitant increase in the membrane fraction (Fig. 5*A*). The level of Pah1 associated with the membrane fraction was detected at a reduced level due to its proteolytic degradation (27). When phosphorylated Pah1 was incubated with membrane fractions overexpressing Nem1-PtA (WT or mutants) and Spo7, the membrane association of Pah1 by the active site mutants was largely comparable to that of the WT control (Fig. 5*B*). This indicated that the active site mutations do not hinder the complex-mediated association of Pah1 with the membrane.

### Effect of Nem1 active site mutations on phenotypes associated with Pah1 function

**Figure 6:**
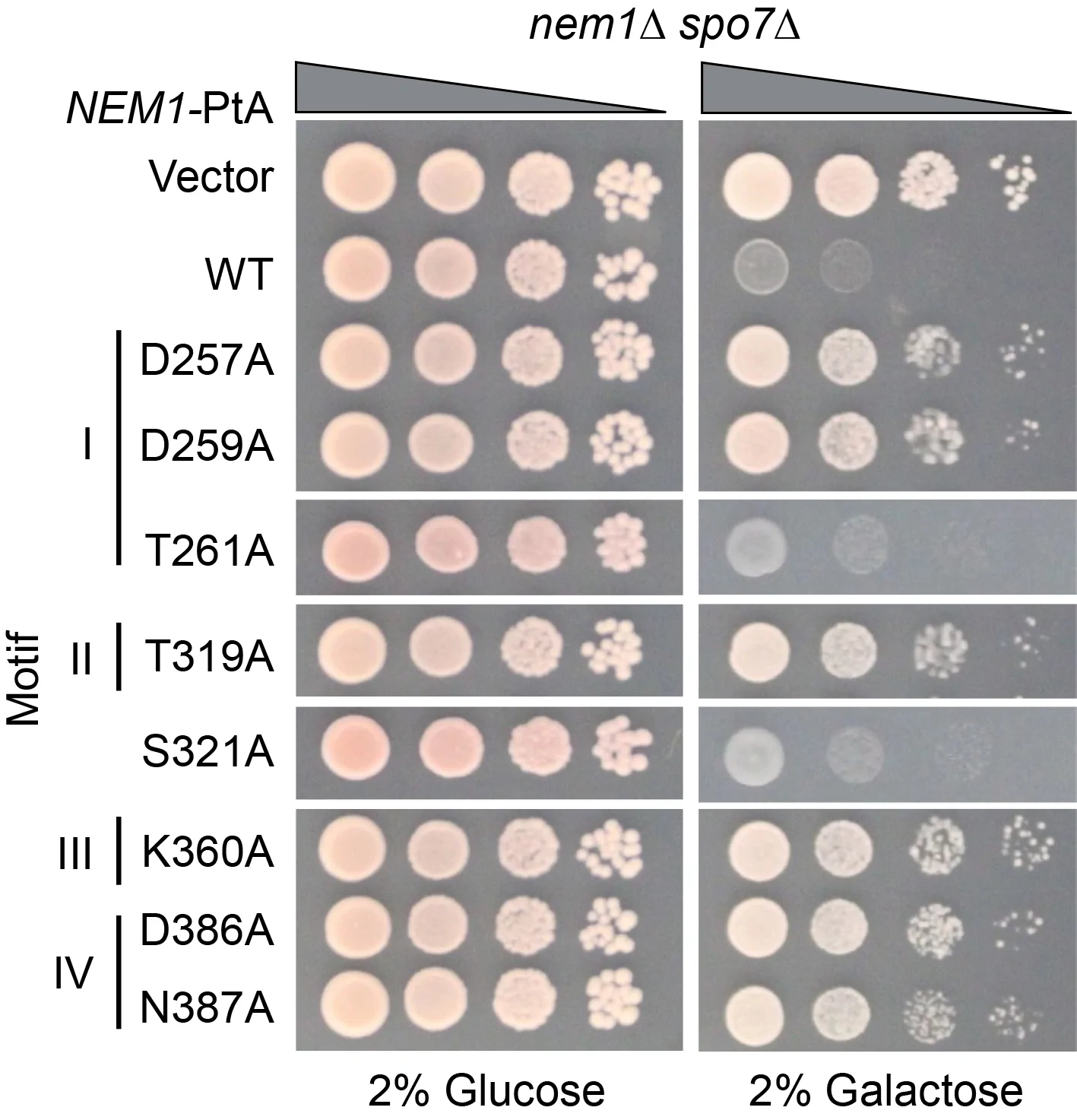
**Nem1 active site mutants rescue the lethality caused by overexpression of the Nem1-Spo7 complex in Pah1-harboring cells.** The *nem1*Δ *spo7*Δ (SS1010) cells were transformed with plasmids YCplac111, YCplac111-*GAL1/10-NEM1*-PtA (WT or mutant derivatives) and pRS314-GAL1/10*-SPO7*. The transformants were grown overnight to saturation in SC-Leu-Trp medium with 2% glucose as carbon source. The cells were washed with water and resuspended in the growth medium containing 2% glucose or 2% galactose. The cell suspension was adjusted to the absorbance of 0.67 at 600 nm, serially diluted (10-fold), and spotted onto SC-Leu-Trp agar plates containing 2% glucose or 2% galactose. Cell growth was scored after incubation for 3 days at 30 °C. The data is representative of five replicate experiments.

Given that the Nem1-Spo7 overexpression is lethal due to the excessive production of active Pah1 (16), the Nem1 active site mutants were analyzed for their effect on cell viability (Fig. 6). As anticipated, no growth was shown by the *nem1*Δ *spo7*Δ cells overexpressing WT Nem1-PtA and Spo7 on galactose medium. In contrast, the overexpression of the Nem1 active site mutants (*e.g.*, D257A, D259A, T319A, K360A, D386A, and N387A) with Spo7 did not cause lethality, indicating a defect in the over-activation of Pah1. The T261A mutation in motif I was the sole exception, as it did not support growth.

**Figure 7:**
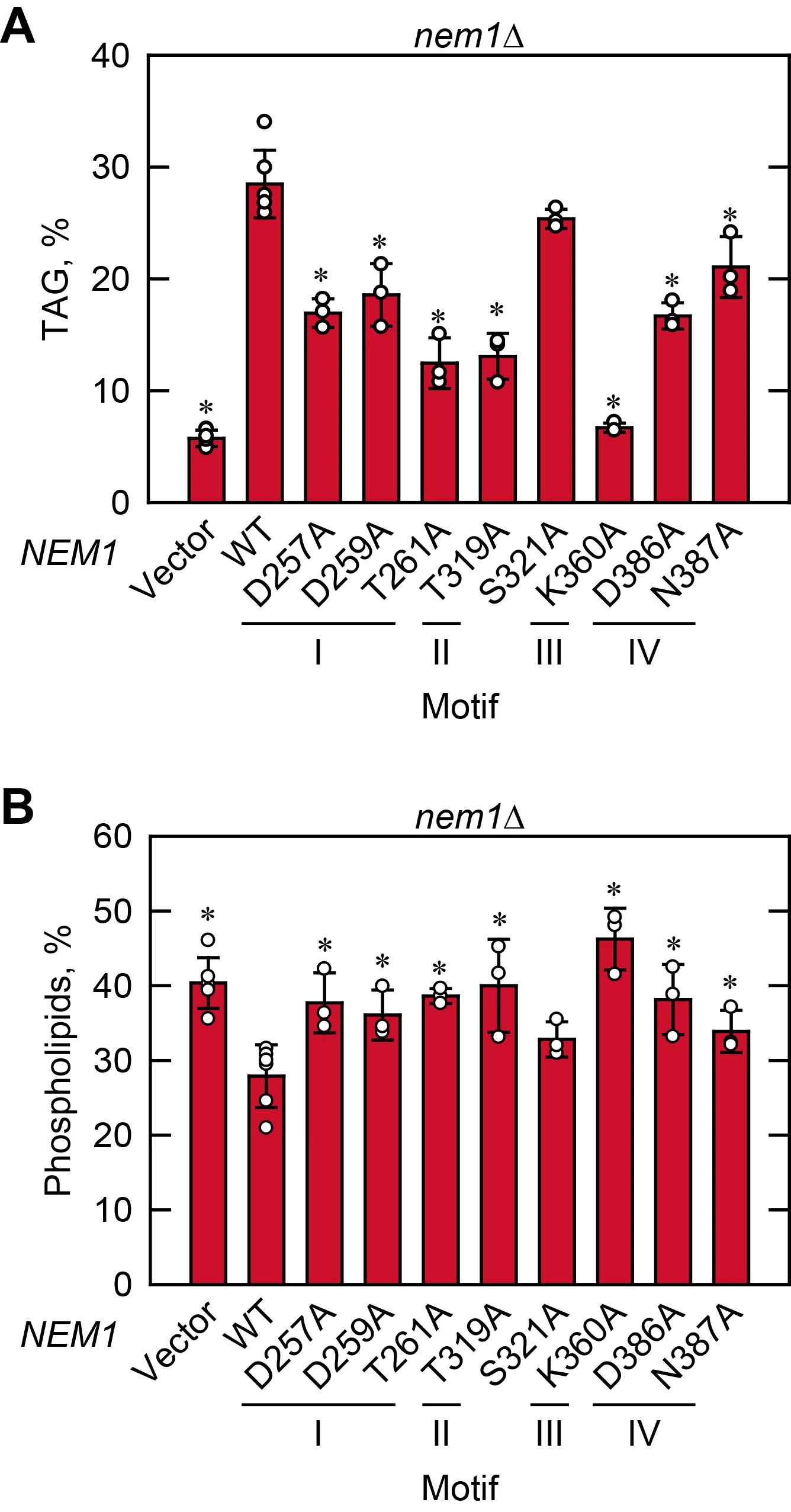
**Effect of the Nem1 active site mutations on the Nem1-Spo7-mediated regulation of the amounts of TAG and phospholipids.** The *nem1*Δ (WMY161) cells expressing plasmids YCplac111 (vector) and YCplac111-*NEM1* (WT or mutant derivatives) were grown to the stationary phase in SC-Leu medium containing [2-^14^C]acetate (1 μCi/ml) at 30 °C. Lipids extracted from the radiolabeled cells were separated by TLC, subjected to phosphorimaging, and quantified by ImageQuant analysis. The levels of TAG (*A*) and phospholipids (*B*) were normalized to total chloroform-soluble lipids. The data are means ± SD (*error bars*) from three independent experiments. The individual data points are also shown. ∗, *p* < 0.05 *versus* WT.

Mutants (*e.g.*, *nem1*Δ, *spo7*Δ, and *pah1*Δ) defective in the Nem1-Spo7/Pah1 phosphatase cascade exhibit a host of phenotypes that include defects in lipid synthesis and aberrant expression of phospholipid synthesis enzymes (1, 13, 32, 34, 58, 59). We examined the ability of the Nem1 active site mutations to complement these phenotypes. *NEM1* and its mutant derivatives were expressed in *nem1*Δ cells under the native promoter to approximate the genomic expression (Table 1). To determine lipid composition, the cells were grown with [2-^14^C]acetate to the stationary phase when TAG levels are highest (1, 13, 14). TLC analysis of radiolabeled lipids showed that in *nem1*Δ cells (vector control), TAG (Fig. 7*A*) and phospholipids (Fig. 7*B*) accounted for 5.7 and 40% of total lipids, respectively. These lipid levels were changed upon expression of the WT *NEM1* allele, which increased TAG content to 28% and decreased phospholipids to 28%. While the active site mutations generally provided some complementation of the *nem1*Δ lipid phenotype, TAG levels remained significantly lower and phospholipid levels significantly higher than those restored by WT *NEM1* (Fig. 7, *A* and *B*). Notably, the K360A mutation exhibited the most severe defect, failing to complement the *nem1*Δ effects on either TAG or phospholipid abundance.

**Figure 8:**
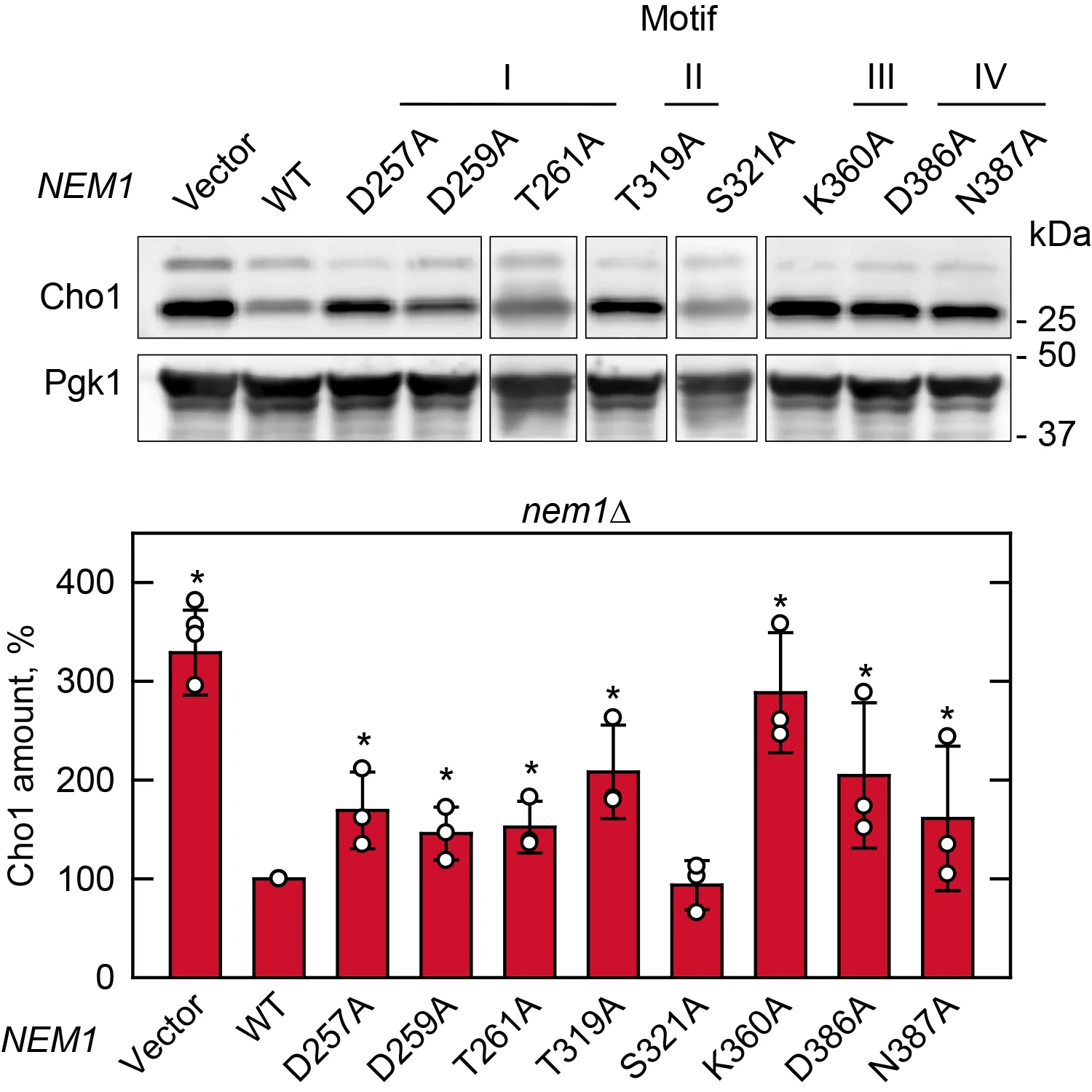
**Effect of the Nem1 active site mutations on the Nem1-Spo7-mediated regulation of Cho1 expression.***Upper*, the *nem1*Δ (WMY161) cells expressing plasmid YCplac111 (vector) and YCplac111-*NEM1* (WT or mutant derivatives) were grown at 30 °C in SC-Leu medium to the stationary phase. Cell extracts (10 μg) were resolved by SDS-PAGE and transferred to a polyvinylidene difluoride membrane, which was then probed for Cho1 and Pgk1 (loading control) with rabbit anti-Cho1 and mouse anti-Pgk1 antibodies, respectively. The positions of Cho1 and Pgk1 are indicated with molecular mass standards. The minor band above the major Cho1 band is a phosphorylated form of the protein (85). T261A, T319A, and S321A samples were electrophoresed on individual gels under identical conditions. The figure incorporates spliced lanes to maintain the positional order of the motif residues. The Western blots are representative of three independent experiments. *Lower*, the relative amount of Cho1 was determined by ImageQuant analysis. The data are means ± SD (*error bars*) from triplicate experiments. The individual data points are shown. ∗, *p* < 0.05 *versus* WT.

The elevation of phospholipid contents in cells lacking the Nem1-Spo7/Pah1 phosphatase cascade stems, at least partially, from the PA-mediated derepression of phospholipid biosynthetic genes containing the UAS_INO_ element (17, 18, 58). This regulation of gene expression is governed by the Henry (Opi1/Ino2-Ino4) regulatory circuit (6, 7, 12, 17, 18, 59, 60). One of the regulated genes that is particularly affected by the phosphatase cascade mutations, which elevate PA levels and disrupt phospholipid synthesis regulation (1, 17), is *CHO1* (58). This gene encodes phosphatidylserine synthase Cho1 (61), the enzyme responsible for the first step in the synthesis of phosphatidylcholine from CDP-DAG (6, 7, 59, 60). During cell growth, Cho1 levels are high in the exponential phase when phospholipid synthesis is active, but lowered in the stationary phase as metabolic flux shifts toward TAG production (32, 34, 58, 59, 62). Consistent with previous findings (32), expressing WT *NEM1* in stationary-phase *nem1*Δ cells reduced the elevated Cho1 levels by approximately three-fold (Fig. 8). In contrast, the active site mutations, most notably K360A, failed to complement fully the *nem1*Δ phenotype, leaving Cho1 expression levels elevated in stationary-phase cells (Fig. 8).

## Discussion

PA phosphatase plays a major role in the control of the PA/DAG balance, and by doing so, the enzyme functions as a “gatekeeper” to direct whether cells synthesize membrane phospholipids or the storage lipid TAG (2, 3). In balancing the lipid intermediates, PA phosphatase also regulates membrane structure, lipid signaling, and the transcription of lipid synthesis genes (2, 3). The Nem1-Spo7 phosphatase complex plays a vital role in the regulation of these processes by governing the phosphorylation state, location, and activation of Pah1 (1, 3, 16, 17, 18, 30, 31). The Nem1-Spo7/Pah1 phosphatase cascade is conserved in higher eukaryotes; the homologous components include the CTDNEP1 (catalytic subunit)-NEP1-R1 (regulatory subunit) complex and the lipin 1 PA phosphatase (63, 64, 65, 66, 67, 68). The importance of the phosphatase cascade to cell physiology is highlighted in yeast, as well as in mice and humans, by a host of cellular defects and lipid-based diseases that are associated with loss of PA phosphatase activity (2, 17, 59, 63, 64, 69, 70, 71, 72, 73, 74, 75, 76, 77, 78, 79, 80). While previous studies have established the importance of the Nem1-Spo7 complex in the recruitment and dephosphorylation of Pah1, the active site of the HAD-like domain remained largely uncharacterized.

The HAD superfamily is defined by four conserved motifs that coordinate Mg^2+^ and facilitate the nucleophilic attack on the phosphate group of the substrate. Our results showed that conserved residues in motifs I-IV are required for the Nem1 catalytic activity. The substitution of these residues with alanine resulted in the loss of Pah1 dephosphorylation. The loss of catalytic activity by the conserved mutations (*i.e.*, D257E and D259E) of motif I aspartates indicates that the geometry and side-chain length of aspartate are strictly required to position the nucleophile and coordinate the Mg^2+^ cofactor.

A key finding of this work is that the catalytic motifs of Nem1 are distinct from the domains required for protein-protein interactions. The co-purification of Spo7 with the Nem1 active site mutants confirms that complex formation does not rely on the catalytic pocket. Furthermore, the Pah1 membrane association assays demonstrated that the catalytically inactive Nem1-Spo7 complex interacts with phosphorylated Pah1 and recruits it to the membrane.

The physiological effects of inactivating Nem1 phosphatase activity were evident in the altered phenotypes associated with Pah1 function. These included rescue of growth inhibition upon Pah1 hyperactivation and altered amounts of lipids and Cho1. A notable exception was the T261A mutation contained within motif I. Although T261A exhibited a defect in dephosphorylating Pah1 *in vitro*, it failed to rescue the growth phenotype upon its overexpression. This raised the suggestion that the galactose-induced overexpressed mutant retains sufficient residual phosphatase activity to sustain the hyperactive Pah1 phenotype. However, despite this implication, we were unable to detect any measurable *in vitro* phosphatase activity, even when the electrophoretic mobility assays were extended to 2 h when compared with the standard 25-min assay.

The TAG and phospholipid levels of the cells expressing the active site mutants were lower and higher, respectively, when compared with the cells expressing the WT *NEM1* allele. This reflects the defect in the Nem1-Spo7 complex-mediated activation of Pah1 function to regulate the bifurcation of PA in lipid synthesis (32, 34, 35). This leads to an accumulation of PA, which in turn regulates the Henry regulatory circuit (12, 17, 18, 58). By sequestering the Opi1 repressor at the membrane, elevated PA levels allow for the constitutive derepression of phospholipid synthetic genes, such as *CHO1* (58). Our observation that the active site mutants, particularly K360A, failed to properly downregulate Cho1 expression in the stationary phase correlates directly with the observed decrease in TAG synthesis. The severity of the K360A phenotype suggests that Motif III may play a particularly critical role in controlling the phosphorylation state of Pah1, structural stability, or substrate positioning of the active site, making it the most detrimental of the mutations tested.

**Figure 9:**
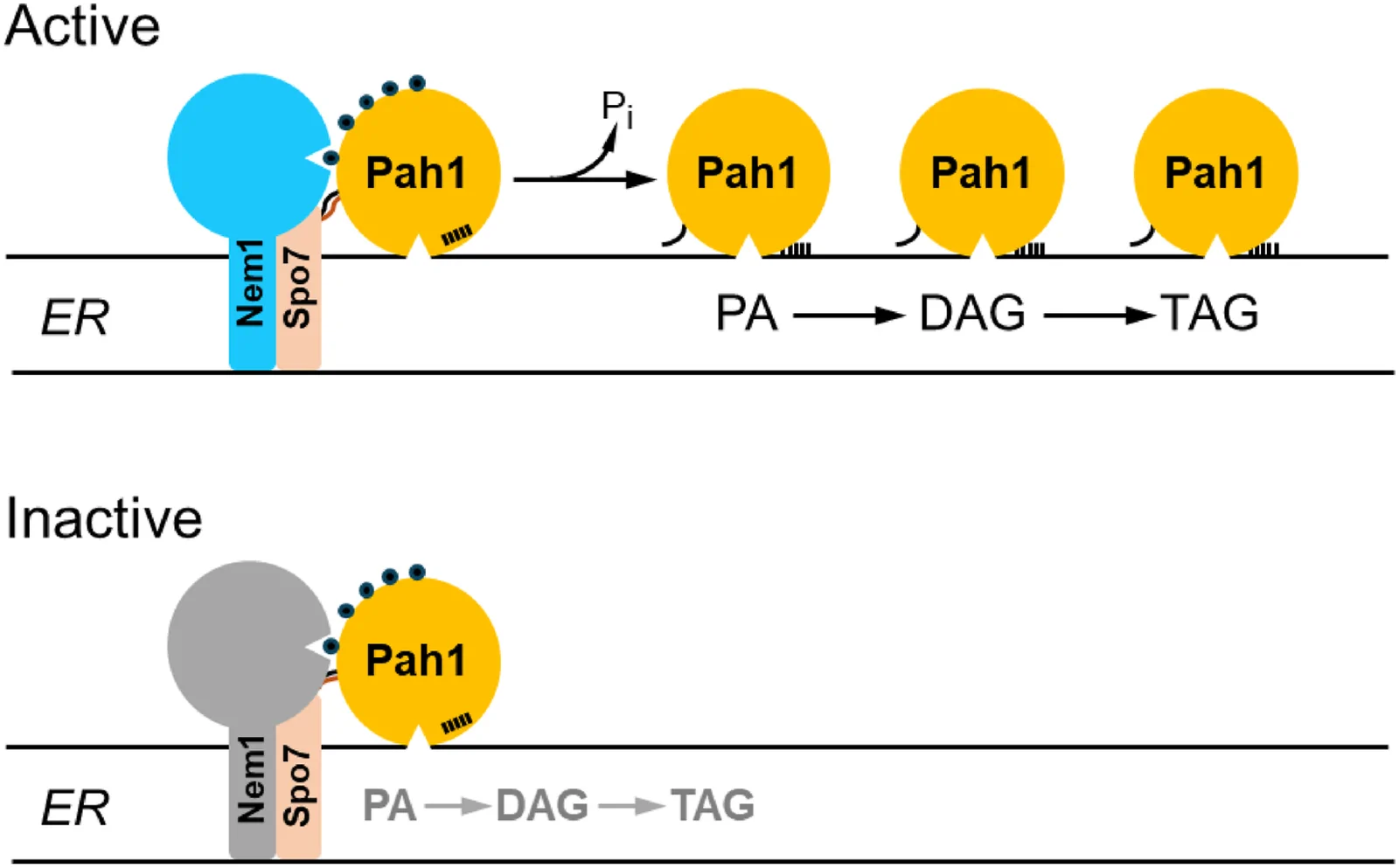
**Model for the activation of Pah1 function by the Nem1-Spo7 complex.***Upper*, phosphorylated Pah1 (*black circles*) associated with the Nem1-Spo7 complex through the acidic tail of Pah1 (*small black line*) and the basic tail of Spo7 (*small orange line*) is dephosphorylated by active Nem1 (*light blue*) at the ER membrane. Following dephosphorylation, Pah1 is released from the complex and associates with the membrane *via* its amphipathic helix (*small black bars*). The active complex produces multiple dephosphorylated Pah1 molecules to catalyze the dephosphorylation of PA to produce DAG for TAG synthesis. *Lower*, Pah1 associated with the complex with inactive Nem1 (*grey*) is not dephosphorylated and released from the complex, but its membrane association allows a low level of PA phosphatase activity (*grey letters*).

Although the active-site mutants displayed defects in lipid synthesis and in the regulation of Cho1 expression, their phenotypes were not as severe when compared with those of the vector control, which lacks Nem1. This indicates that the mutations produce a “leaky” loss of Pah1 function. How might this occur if the active site mutants are unable to dephosphorylate and activate Pah1? As noted above, these mutations do not disrupt formation of the Nem1-Spo7 complex or its ability to recruit Pah1 to membranes. We propose a model whereby phosphorylated Pah1 is recruited to the membrane by the catalytically inactive Nem1-Spo7 complex, allowing a limited level of membrane association and Pah1 activity to persist (Fig. 9). This model also provides an alternative explanation for the unexpected growth phenotype imparted by the T261A mutation, especially if more Pah1 is recruited to the complex in cells expressing the mutant enzyme (Fig. 5*B*). Parenthetically, phosphorylation *per se* does not totally inactivate PA phosphatase activity (18, 19, 21, 26).

Like Nem1, Pah1 performs its phosphatase activity through an HAD-like catalytic domain containing active site motifs I–IV (40). Despite this shared catalytic framework, the two enzymes differ in their active-site motifs and their modes of regulation. In Nem1, motif II consists of T/S (Fig. 2), whereas in Pah1 the motif is expanded to T/S*X*R (40). The Arg residue in this motif is critical for PA phosphatase activity (40, 81). Motif IV also differs between the two phosphatase enzymes: Nem1 contains D(D/N) (Fig. 2), while Pah1 possesses G(D/N)*XXX*D (40).

Nem1 requires the regulatory subunit Spo7 for activity (16, 33, 34, 35), whereas Pah1 operates without any regulatory partner (1). Spo7 is essential for recruiting Pah1 for Nem1 to act on (35). Through this recruitment, Spo7 not only ensures that the complex specifically targets Pah1 but also positions Pah1 at the PA-containing ER membrane, thereby facilitating efficient production of DAG for its conversion into TAG. In the absence of Spo7, Nem1 is unable to dephosphorylate Pah1 (34, 35). The mammalian homolog of Nem1, CTDNEP1, dephosphorylates lipin 1 (65, 66, 67, 68). Although CTDNEP1 can act on lipin 1 phosphopeptide substrates without its Spo7-like partner NEP1-R1 (82), the CTDNEP1–NEP1-R1 complex is required for dephosphorylation of full-length lipin 1 (65, 66, 67, 68). Whether Nem1 can dephosphorylate Pah1 phosphopeptide substrates in the absence of Spo7 remains unknown.

Although Pah1 lacks a regulatory subunit, its structure contains an N-LIP domain that is conserved among Pah1/lipin-1 homologs (1, 39, 83). In contrast, non-Pah1/lipin HAD-like phosphatases such as Nem1 do not contain this domain (40). Direct experimental evidence defining the specific contribution of the N-LIP domain to Pah1 catalytic activity is still lacking, but it is known that Gly-80, located within the N-LIP domain, is essential for PA phosphatase activity *in vitro* and *in vivo* (84). The corresponding N-LIP domain residue in mammalian lipin 1, Gly-84, is likewise required for its function (83). Another proposed role of the N-LIP domain is to facilitate membrane targeting of Pah1 for PA association. This membrane association depends on an amphipathic helix (29), which forms an N-terminal extension of the N-LIP domain (Fig. 1).

**Figure 10:**
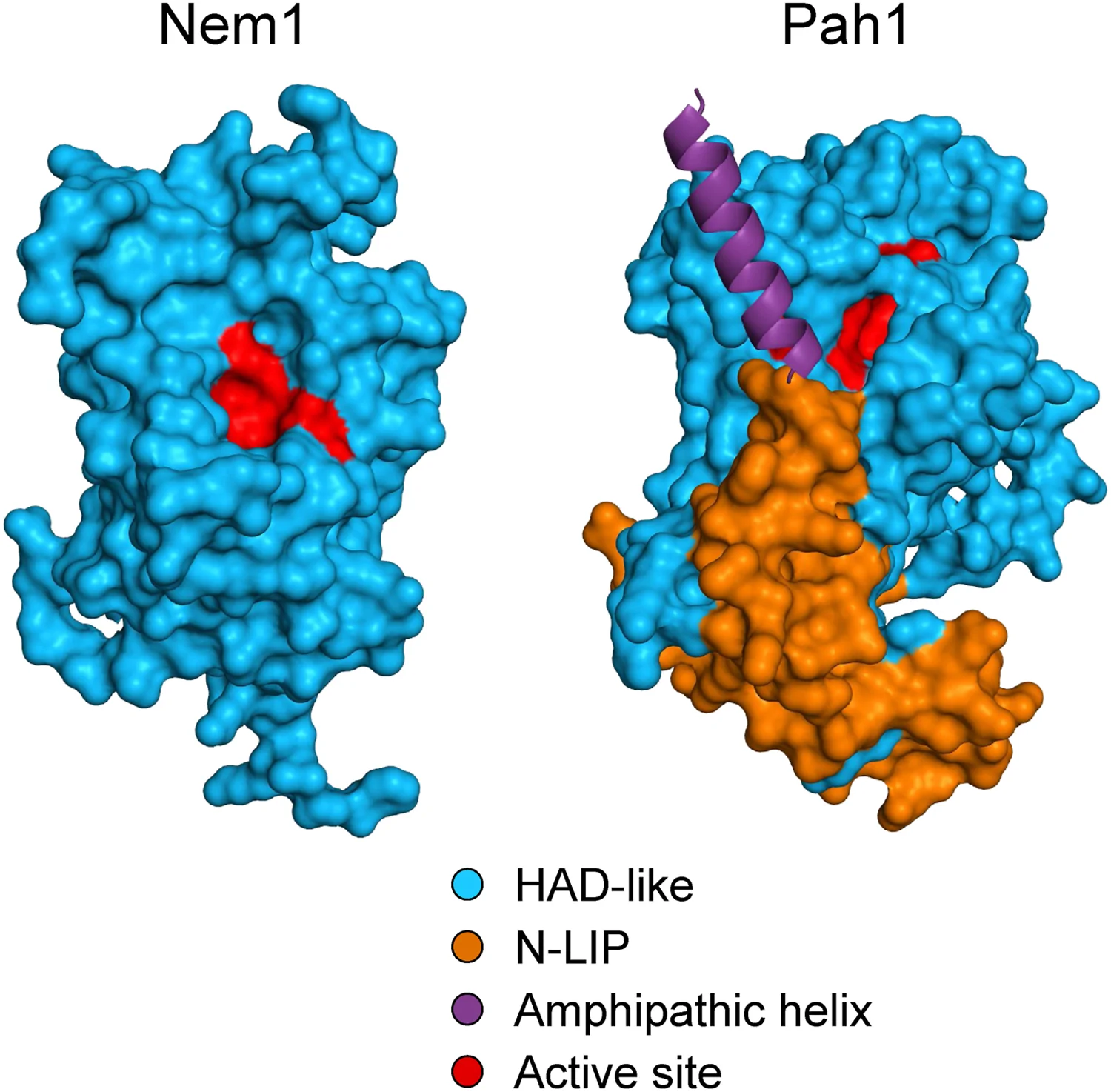
**Predicted structures of Nem1 and Pah1 reveal a difference in the active site size.** The surface views of Nem1 and Pah1 predicted by AlphaFold (56) are visualized by the PyMol program. The domains/regions of each protein are indicated in the legend.

A key distinction between the substrates of Nem1 and Pah1 is that Nem1 acts on Pah1, a large water-soluble protein, whereas Pah1 acts on PA, a relatively small membrane-associated lipid. AlphaFold/PyMol surface representations of the Nem1 and Pah1 catalytic cores predict that Nem1 possesses a larger catalytic cavity (43.5 Å^2^) than Pah1 (31.7 Å^2^) (Fig. 10). This larger cavity is consistent with accommodating phosphoamino acids of the protein substrate Pah1, while the smaller cavity predicted for Pah1 is suited for binding phosphate of its substrate PA.

In conclusion, this work establishes the residues within the four conserved motifs contained in the active site of the HAD-like domain as the functional center of Nem1-mediated dephosphorylation of Pah1. By defining these catalytic requirements, we further understand how the Nem1-Spo7 complex acts as a metabolic switch, converting PA from a signaling molecule and phospholipid precursor into a source of storage lipids.

## Experimental procedures

### Reagents

All chemicals were reagent grade or better. Growth media were from Difco Laboratories. Primers for PCR site-directed mutagenesis were prepared by Sigma-Aldrich. DNA- and plasmid-extraction kits were sourced from Qiagen. Carrier DNA was from Clontech. Enzyme reagents for DNA manipulation and the Q5 site-directed mutagenesis kit came from New England Biolabs. Protein molecular mass standards and the Bradford protein assay reagent were obtained from Bio-Rad. Polyvinylidene difluoride membranes, enhanced chemifluorescence substrate for immunoblotting, Protein A-Sepharose CL-4B, and IgG-Sepharose beads were purchased from GE Healthcare. Lipids were purchased from Avanti Polar Lipids. Silica Gel 60 F254 plates, ampicillin, bovine serum albumin, β-mercaptoethanol, nucleotides, Triton X-100, and protease inhibitors were purchased from Millipore-Sigma. EDTA-free cOmplete ULTRA protease inhibitor tablets were from Roche. [2-^14^C]acetate and Ecoscint O scintillation counting solution were purchased from Revvity and National Diagnostics, respectively.

### Antibodies

Rabbit anti-protein A antibody (product number P3775, lot 025K4777) and goat anti-mouse IgG antibody conjugated with alkaline phosphatase (product number A3562, lot number SLBG1482V) were purchased from Millipore-Sigma. Mouse anti-Pgk1 antibody (product number ab113687, lot number 2101050637) and goat anti-rabbit IgG antibody conjugated with alkaline phosphatase (product number 31340, lot number NJ178812) were sourced from Abcam and Thermo Fisher Scientific, respectively. Rabbit polyclonal antibodies were generated against Spo7 sequence EDDLRRQAHEQK (residues 58-69) (34), Pah1 sequence TSIDKEFKKLSVSKAGA (residues 778-794) (20), and Cho1 sequence MVESDEDFAPQEFPH (residues 1-15) (85). The IgG fraction of the antisera was purified by affinity chromatography with protein A-Sepharose (86).

### Strains and growth conditions

The bacterial and yeast strains used in this study are listed in Table 1. *Escherichia coli* strain DH5α was used for the propagation of plasmids. The *S. cerevisiae nem1*Δ mutant (WMY161) was used for the low-copy expression of *NEM1* (WT and mutants). The *nem1*Δ *spo7*Δ mutant (SS1010) was used to examine the lethal effect of overexpressing *NEM1* (WT and mutants) and *SPO7* (17). The *nem1*Δ *spo7*Δ *pah1*Δ mutant (GHY85) was used for the overexpression of *NEM1* (WT and mutants) and *SPO7* without the lethal effect caused by the expression of *PAH1* (17). The *pah1*Δ *nem1*Δ mutant (SS1132) was used for the overexpression and purification of the hyperphosphorylated form of Pah1 (17, 57).

*E. coli* and *S. cerevisiae* cells were cultured by conventional methods (87). *E. coli* transformants were grown at 37 °C in Luria-Bertani broth medium (1% tryptone, 0.5% yeast extract, 1% NaCl, pH 7.0) containing 100 μg/ml ampicillin. Yeast cells were grown at 30 °C in YPD medium (1% yeast extract, 2% peptone, and 2% glucose) or synthetic complete (SC) medium containing 2% glucose. The plasmid-containing cells were grown in synthetic dropout media (SC-Leu or SC-Leu-Trp). Cell growth in liquid medium was estimated by measuring the absorbance of liquid cultures at 600 nm (A_600_). For lipid analysis, *nem1*Δ cells expressing *NEM1* (WT and mutants) were grown in synthetic dropout media to the exponential phase (A_600_ ∼ 0.5), and then grown for an additional 24 h to the stationary phase. For galactose induction of *GAL1/10*-*NEM1*-PtA (WT and mutants) and *SPO7*, cells were inoculated at a final cell density of A_600_ ∼ 0.1 and grown overnight in SC-Leu-Trp (2% glucose) to saturation. The saturated culture was harvested by centrifugation at 1500*g* for 10 min, washed with water, and resuspended in SC-Leu-Trp (2% galactose and 1% raffinose) media at a final cell density of A_600_ ∼ 0.4 and incubated for 14 h (A_600_ ∼ 0.4-0.5) with shaking at 250 rpm. For lethality spot tests, yeast transformants were grown to saturation in SC-Leu-Trp (2% glucose), collected by centrifugation, washed with water, resuspended in SC-Leu-Trp to a cell density of A_600_ ∼ 0.67, serially diluted (10-fold), and spotted onto SC-Leu-Trp agar plates containing 2% glucose or galactose. Growth was scored after incubation for 3 days at 30 °C.

### Plasmids and DNA manipulations

The plasmids used in this study are listed in Table 1. YCplac111 (88) and pRS314 (89) are low-copy *E. coli*/*S. cerevisiae* shuttle vectors with *LEU2* and *TRP1*, respectively. YCplac111-*NEM1* directs the low-copy expression of *NEM1* from its native promoter (32), whereas YCplac111-*GAL1/10*-*NEM1*-PtA directs the overexpression of *NEM1*-PtA from the *GAL1/10* promoter (16). pRS314-*GAL1/10-SPO7* was used for the overexpression of *SPO7* (38). pGH452 bearing *PAH1*-PtA under the control of *GAL1/10* promoter was used for the purification of phosphorylated Pah1 (57). The derivatives of YCplac111-*NEM1* and YCplac111-*GAL1/10*-*NEM1*-PtA containing *NEM1* mutations in active site motifs I-IV were constructed by PCR-mediated site-directed mutagenesis (33). All mutations were confirmed by DNA sequencing. The isolation of plasmid DNA, PCR amplification, restriction enzyme digestion, and DNA ligation were performed by standard methods (87, 90, 91). Plasmid transformations of *E. coli* (90) and *S. cerevisiae* (92) were performed by standard methods.

### Preparation of cell extracts, membranes, and enzymes

All steps were conducted at 4 °C. *S. cerevisiae* cultures were harvested by centrifugation at 1500*g* for 10 min. Cells were washed with water and resuspended in lysis buffer (50 mM Tris-HCl (pH 7.5), 10% glycerol, 10 mM 2-mercaptoethanol, 1 mM EDTA, 0.5 mM phenylmethylsulfonyl fluoride, 1 mM benzamidine, 5 μg/ml aprotinin, 5 μg/ml leupeptin, and 5 μg/ml pepstatin). Cells were lysed by mechanical disruption with glass beads (0.5-mm diameter) using a BioSpec Products Mini-Beadbeater-16 (five repeats of 1-min bursts, followed by 2-min cooling) (93). Lysates were centrifuged at 1500*g* for 10 min to separate unbroken cells and cell debris (pellet) from the cell extract (supernatant). The total membrane fraction was collected from the cell extract by centrifugation at 100,000*g* for 1 h (93); the membranes were suspended in lysis buffer prepared without EDTA.

The Protein A-tagged Nem1-Spo7 complex was isolated from cell lysates (3 mg protein) of *nem1*Δ *spo7*Δ *pah1*Δ (GHY85) cells expressing plasmids YCplac111-*GAL1/10*-*NEM1*-PtA (WT or mutants) and pRS314-*GAL 1/10-SPO7* by affinity chromatography using IgG-Sepharose (16, 30). The isolated Nem1-PtA and Spo7 proteins were detected by Western blotting using anti-protein A and anti-Spo7 antibodies, respectively. The amounts of the complex proteins were not sufficient for detection by Coomassie Blue protein staining on an SDS-polyacrylamide gel. Phosphorylated Pah1 was purified to near homogeneity from the *pah1*Δ *nem1*Δ mutant cells expressing plasmid pGH452 using a combination of IgG-Sepharose chromatography, anion exchange chromatography, and size exclusion chromatography (57). All protein preparations were stored at −80 °C.

### SDS-PAGE and Western blotting

SDS-PAGE (94) using 12.5%, 10%, or 6% polyacrylamide gels and Western blotting (95, 96) were performed by standard protocols. Polyacrylamide gel loading was normalized by protein amount as determined by the method of Bradford (97) using bovine serum albumin as a standard. Protein loading and transfer from the polyacrylamide gel to the polyvinylidene difluoride membranes was confirmed by Ponceau S staining. Polyvinylidene difluoride membranes were probed with rabbit anti-protein A (1 μg/ml), rabbit anti-Spo7 (2 μg/ml), rabbit anti-Pah1 (1 μg/ml), rabbit anti-Cho1 (0.27 μg/ml), or mouse anti-Pgk1 (0.1 μg/ml) antibody. The secondary alkaline phosphatase-conjugated goat anti-rabbit IgG antibody and goat anti-mouse IgG antibody were used at dilutions of 1:1500 and 1:3,000, respectively. Immune complexes were detected with the enhanced chemifluorescence substrate for alkaline phosphatase. Fluorescence signals from immunoblots were acquired by a Storm 860 Molecular Imager, and signal intensities were analyzed by the ImageQuant TL software (GE Healthcare).

### Nem1-Spo7 protein phosphatase assay

The Nem1-Spo7 protein phosphatase activity was determined by the ability of the enzyme to dephosphorylate and increase the electrophoretic mobility of Pah1 (30). The reaction mixture in a total volume of 20 μl contained 100 mM sodium acetate (pH 5.0), 10 mM MgCl_2_, 1 mM DTT, 5 ng Pah1, and 20 μg membranes containing overexpressed Nem1 (WT and mutants) and Spo7 (30). Following a 25-min incubation, samples were subjected to SDS-PAGE with a 6% polyacrylamide gel, followed by Western blotting with anti-Pah1 antibody (33, 34, 35). The densitogram of Pah1 along its migration was analyzed by the line graph function of ImageQuant software.

### Lipid labeling and analysis

*S. cerevisiae* cells were labeled to steady state [2-^14^C]acetate (98). Following growth to stationary phase, lipids were extracted from the radiolabeled cells by the method of Bligh and Dyer (99). The lipids in the chloroform extract were separated by one-dimensional TLC using hexane/diethyl ether/glacial acetic acid (40:10:1, v/v) as the solvent system (100). The resolved lipids on silica gel 60 plates were visualized by phosphorimaging with the Storm 860 Molecular Imager (GE Healthcare), and the amounts of TAG and phospholipids relative to the total lipid extract were quantified by ImageQuant software using a standard curve of [2-^14^C]acetate. The identity of radiolabeled TAG and phospholipids on the TLC plate was confirmed by co-migration of authentic standards visualized by charring following spray with sulfuric acid (101).

### Predicted structure analysis

AlphaFold (56, 102, 103) predicted structures of the *S. cerevisiae* Nem1-Spo7 complex were visualized by the PyMol program.

### Data analysis

Statistical analyses of data using the t-test were performed with Microsoft Excel software. *p* values < 0.05 were taken as a significant difference.

## Data availability

All data are contained within the manuscript.

## Conflict of interest

The authors declare that they have no conflicts of interest with the contents of this article.
